# Antioxidant and Anti-Inflammatory Effects of *Opuntia* Extracts on a Model of Diet-Induced Steatosis

**DOI:** 10.3390/antiox13111416

**Published:** 2024-11-19

**Authors:** Irene Besné-Eseverri, María Ángeles Martín, Gloria Lobo, M. Pilar Cano, María P. Portillo, Jenifer Trepiana

**Affiliations:** 1 Nutrition and Obesity Group, Department of Nutrition and Food Sciences, Faculty of Pharmacy, University of the Basque Country (UPV/EHU) and Lucio Lascaray Research Centre, 01006 Vitoria-Gasteiz, Spain; irene.besne@ehu.eus (I.B.-E.); mariapuy.portillo@ehu.eus (M.P.P.); 2 CIBER Physiopathology of Obesity and Nutrition (CIBERobn), Institute of Health Carlos III, 28029 Madrid, Spain; 3 Science and Food Technology and Nutrition Institute (ICTAN-CSIC), 28040 Madrid, Spain; amartina@ictan.csic.es; 4 CIBER Diabetes and Related Metabolic Diseases (CIBERdem), Institute of Health Carlos III, 28029 Madrid, Spain; 5 Department of Crop Production in Tropical and Subtropical Areas, Instituto Canario de Investigaciones Agrarias (ICIA), 38297 Tenerife, Spain; globo@icia.es; 6 Laboratory of Phytochemistry and Plant Food Functionality, Biotechnology and Food Microbiology Department, Institute of Food Science Research (CIAL) (CSIC-UAM), Nicolás Cabrera 9, 28049 Madrid, Spain; mpilar.cano@csic.es; 7 BIOARABA Institute of Health, 01009 Vitoria-Gasteiz, Spain

**Keywords:** MAFLD, *Opuntia ficus-indica*, *Opuntia stricta* var. *dillenii*, oxidative stress, inflammation, apoptosis, DNA damage, liver, steatosis, rat

## Abstract

Oxidative stress and inflammation are widely recognised as factors that can initiate and facilitate the development of MAFLD. The aim of this study is to analyse the effect of low and high doses of *Opuntia stricta* var. *dillenii* peel extract (L-OD and H-OD, respectively) and *Opuntia ficus-indica* var. *colorada* pulp extract (L-OFI and H-OFI, respectively), which are rich in betalains and phenolic compounds, on oxidative stress, inflammation, DNA damage and apoptosis in rat livers with diet-induced steatosis. Steatotic diet led to increased final body and liver weight, serum transaminases, hepatic TG content, oxidative status and cell death. H-OFI treatment decreased serum AST levels, while L-OFI reduced hepatic TG accumulation. Oxidative stress was partially prevented with H-OD and H-OFI supplementation, and pro-inflammatory cytokines levels were especially improved with H-OFI treatment. Moreover, H-OFI appears to prevent DNA damage markers. Finally, H-OD and L-OFI supplementation down-regulated the apoptotic pathway. In conclusion, both H-OD and H-OFI supplementation were effective in regulating the progression to metabolic steatohepatitis, triggering different mechanisms of action.

## 1. Introduction

Metabolic dysfunction-associated fatty liver disease (MAFLD) is the leading cause of chronic liver disease, with a global prevalence of approximately 30% [1]. It is characterised by excessive hepatic fat accumulation (≥5% of hepatic steatosis) in the absence of alcohol consumption, steatogenic medications, or hereditary disorders [2]. MAFLD is a term that encompasses a wide spectrum of alterations, ranging from simple steatosis to metabolic steatohepatitis, which can lead to MAFLD-related cirrhosis and hepatocellular carcinoma (HCC) [2,3].

Lifestyle represents the most relevant factor in the development of MAFLD. In this context, high-fat diets were initially considered the dietary pattern responsible for obesity and its associated metabolic complications. Recently, more attention has been paid to the detrimental effects of sugar, especially fructose. Fructose is a highly lipogenic nutrient, acting both as a substrate for lipogenesis and as an inducer of the lipogenic pathway. In fact, experimental designs involving animals fed a high-fructose high-fat diet are valuable tools to analyse the steatogenic effect caused by such diets, as they reflect the “Western diet” commonly followed by the population [4]. The development of MAFLD depends on multiple cumulative insults, including a non-modifiable genetic susceptibility, as described by the “multiple-hit” hypothesis” [5,6]. According to this proposition, fat accumulation in the liver is induced after the “first hit”, leading to inflammation and cell death (apoptosis), which is mediated by oxidative stress—the “second hit”. Indeed, oxidative stress is regarded as the primary factor in liver injury and disease progression [6].

Oxidative stress is described as an imbalance between the production of reactive oxygen and nitrogen species (RONS) and the antioxidant scavenging capacity of the host in favour of the former. Some of the RONS produced in cell metabolism are free superoxide anion radicals (O_2_^−^), nitric oxide (NO), hydroxyl radicals (**^.^**OH) and hydrogen peroxide (H_2_O_2_) [5]. Scavenging enzymes, such as superoxide dismutase (SOD), catalase (CAT) and glutathione peroxidase (GPx), react with oxidants and remove the free radicals and reactive species to reduce their oxidation capacity. Free radicals can also be scavenged by electron donors, such as reduced glutathione (GSH), which reduces oxidised glutathione (GSSG) by using NADPH [7].

Approximately 20% of the individuals with MAFLD have metabolic steatohepatitis, which is characterised by chronic liver inflammation, low-grade systemic inflammation and oxidative stress. This pathology is considered complex and multifactorial and may involve the activation of pro-inflammatory cascades, including inflammatory cytokines and inflammasomes, such as the NLR family pyrin domain-containing 3 (NLRP3) [8].

Although lifestyle interventions have been proven beneficial in the management of MAFLD, achieving these changes can be challenging due to low adherence. Moreover, there is currently no approved pharmacological treatment for this disease [9]. Given the relationship between oxidative stress and MAFLD progression, significant attention has been directed towards bioactive compounds present in foods or plants with antioxidant properties. *Opuntia* (from the *Cactaceae* family) grows wild in arid and desert environments and serves as an important source of bioactive compounds, such as betalains (betacyanins and betaxanthins), phenolic compounds (flavonoids and phenolic acids), carotenoids, vitamins and fibre. The composition of these components can vary depending on the part of the plant [10,11]. Promising results have been reported in studies using extracts or products derived from various *Opuntia* species for liver health, although most studies have focused on *Opuntia ficus-indica* species and employed chemicals to generate liver damage [12]. *Opuntia stricta* var. *dillenii*, a rarely studied species, grows wild in various regions, including Spain (Canary Islands, Murcia and Almeria), Italy, India and Africa, among others. It is characterised by its betalain content, with betanin being the most abundant pigment, responsible for the fruit’s purple colour [13,14]. In a prior study conducted by our research group, we examined the effects of whole fruit, pulp, peel and bagasse extracts on the prevention of triglyceride accumulation in an in vitro model of hepatic steatosis. Our results confirmed that the most effective extracts were from *Opuntia ficus-indica* var. *colorada* pulp and *Opuntia stricta* var. *dillenii* peel (data submitted).

In this scenario, the aim of this research is to analyse the effectiveness of *Opuntia ficus-indica* var. *colorada* pulp extract and *Opuntia stricta* var. *dillenii* peel extract in the prevention of oxidative stress, inflammation and apoptosis in a murine model of diet-induced liver steatosis.

## 2. Materials and Methods

### 2.1. Opuntia stricta var. dillenii and Opuntia ficus-indica var. colorada Extracts

In a previous study by our group, we analysed the anti-steatotic effects of several extracts from *Opuntia ficus-indica* fruits (pulp or peel) of different varieties (*colorada*, *sanguinos* and *pelota*) and extracts from *Opuntia stricta* var. *dillenii* (whole fruit, pulp, peel and bagasse) on AML-12 hepatocytes [15]. According to the results obtained, the two most efficient extracts were the peel of *Opuntia stricta* var. *dillenii* and the pulp of *Opuntia ficus-indica* var. *colorada*. Therefore, these extracts were selected for the present in vivo study.

Prickly pears of *Opuntia stricta* var. *dillenii* were collected from Tenerife (28°32′03″ N, 16°23′50″ W above sea level) in the Canary Islands, Spain, along with prickly pears of the Spanish orange *Colorada* variety from Fasnia (Tenerife, Canary Islands, Spain; 28°14′44″ N, 16°26′10″ W; 446 m above sea level) [16].

Aqueous prickly pear extracts were obtained from freeze-dried tissues by repeatedly extraction with methanol–water (1:1, *v*:*v*) and methanol to obtain extracts rich in betalains and phenolic compounds [17]. The extracts were freeze-dried, and a stock solution of 200 mg/mL (dissolved in water) was prepared for each extract and aliquoted for storage at −20 °C until administration to the animals.

As shown in Table 1, although both *Opuntia* varieties are rich in betalains and phenolic compounds, there are significant differences among them. The peel of *Opuntia stricta* var. *dillenii* is characterised by a high content of betanin, isobetanin, phyllocactin and neobetanin (betalains), as well as piscidic acid (phenolic acid). In contrast, the pulp of *Opuntia ficus-indica* var. *colorada* is rich in indicaxanthin (betalain) and piscidic acid (phenolic compound). Although in smaller amounts, other betaxanthins are also found in this extract.

### 2.2. Animals, Diets and Experimental Design

Sixty male Wistar rats (4-week-old; 125–145 g) were purchased from Envigo (Barcelona, Spain) to conduct the experiment, which was performed in accordance with the institution’s guidelines for the care and use of laboratory animals (Ethical code: M20_2022_283). Rats were housed in pairs in polycarbonate cages in an air-conditioned room (22 ± 2 °C) with a 12 h light–dark cycle. After a one-week adaptation period, the animals were assigned to six different experimental groups. The control group (C group; n = 10) was fed a standard commercial diet (D10012G; Research Diets, New Brunswick, NJ, USA), and the rest of the animals were fed a high-fat high-fructose diet (HFHF; D21052401; Research Diets, New Brunswick, NJ, 113 USA) (Table 2). The rats fed with HFHF diet received either the diet alone (HFHF group; n = 10) or supplemented with the *Opuntia* extracts in an oral solution containing 2.5% sucrose along with the appropriate amount of each extract to achieve either a low dose of 25 mg/kg body weight (L-OD group; n = 10; L-OFI group; n = 10) or a high dose of 100 mg/kg body weight (H-OD group; n = 10; H-OFI group; n = 10). The dose choice was based on the reported literature, where most of the authors supplemented rodents with doses between 25 and 300 mg/kg/d of Opuntia extracts, the dose of 100 mg/kg/d being the most commonly used [12]. The rats not treated with the extracts (C and HFHF groups) only received the solution containing 2.5% sucrose as a vehicle. The treatments and the vehicle were administered orally daily by using a plastic Pasteur pipette. Animals had free access to food and water, and food intake and body weight were measured on a daily basis. Regarding the food intake, a manual weighing of the food dish before and after the feeding period (each 24 h) was carried out. The treatment period lasted eight weeks.

Food was removed 12 h before the end of the experimental period. The last treatment was administered 3 h prior to euthanasia of the animals under anaesthesia (chloral hydrate) by cardiac exsanguination. The liver was dissected, weighted and immediately frozen in liquid nitrogen. Serum was obtained from blood samples after centrifugation (1000× *g*, 10 min and 4 °C). All samples were stored at −80 °C until analysis.

### 2.3. Liver Triglyceride Content and Serum Transaminases Determination

The extraction of total hepatic lipids was performed following the method described by Folch et al. [18]. The lipid extract was then dissolved in isopropanol, and the triacylglycerol content was assessed by spectrophotometry using a commercial kit (Spinreact, Barcelona, Spain). Serum levels of alanine aminotransferase (ALT) and aspartate aminotransferase (AST) were measured using commercial kits (Biosystems, Barcelona, Spain).

### 2.4. Serum Uric Acid Concentration and C-Reactive Protein Determination

Serum uric acid was determined with a spectrophotometric commercial kit (Spinreact, Barcelona, Spain), while serum C-reactive protein (CRP) levels were measured using an enzyme-linked immunosorbent assay (ELISA) kit (Sigma-Aldrich, St. Louis, MO, USA).

### 2.5. Liver Homogenates and Total Protein Determination

Liver samples (100 mg) were homogenised in 1.5 mL of PBS (0.15 M NaCl, 3 mM KCl, 3 mM NaH_2_PO_4_, 7.5 mM Na_2_HPO_4_; pH 7.4) containing protease inhibitors (1 mM phenylmethylsulphonyl fluoride and 0.1 mM iodoacetamide). The homogenates were centrifuged at 800× *g* for 5 min at 4 °C. These lysates were used for all subsequent determinations and for immunoblotting. Total protein concentration in the homogenates was then spectrophotometrically quantified at 595 nm by Bradford assay [19] using bovine serum albumin as standard.

### 2.6. Measurement of the Parameters Related to Oxidative Stress in Liver

#### 2.6.1. Lipid Peroxidation Measurement

Rat liver samples were homogenised, and lipid peroxidation was determined using a commercial TBARS assay kit (Cayman Chemical, Ann Arbor, MI, USA). This method measures Thiobarbituric Acid Reactive Substances (TBARS) as a marker of lipid peroxidation, involving the reaction of malondialdehyde (MDA) and thiobarbituric acid (TBA) under high temperature and acidic conditions. The MDA–TBA adduct was detected using an Infinite 200Pro plate reader (Tecan, Männedorf, Zurich, Switzerland). Results were expressed as µM MDA/mg protein.

#### 2.6.2. Protein Carbonyl Determination

Protein carbonyl content in rat liver lysates was measured using a commercial kit (Sigma-Aldrich, St. Louis, MO, USA). Carbonyl levels were determined by the derivatisation of protein carbonyl groups with 2,4-dinitrophenyl-hydrazine (DNPH), resulting in the formation of dinitrophenyl (DNP) hydrazone adducts, which were identified spectrophotometrically in an Infinite 200Pro plate reader (Tecan, Männedorf, Zurich, Switzerland). Final protein carbonyl values were expressed in nmol carbonyl/mg protein.

#### 2.6.3. Total Antioxidant Capacity Determination

Rat liver homogenates were utilised to assess total antioxidant capacity using the OxiSelect Oxygen Radical Antioxidant Capacity (ORAC) activity assay commercial kit (Cell Biolabs, San Diego, CA, USA). The ORAC assay involved the application of fluorescein as a fluorescent probe. The free radical initiator AAPH (2,2-azobis [2-amidinopropane] dihydrochloride) was employed to generate peroxyl radicals. AAPH was subsequently added to the sample, and the fluorescence was reordered using an Infinite 200Pro plate reader (Tecan, Männedorf, Zurich, Switzerland). Additionally, a calibration curve was constructed using a Trolox solution. Lastly, calculations were conducted based on differences in areas under the fluorescence decay curve among the blank, samples and standards. Final ORAC values were reported as µM Trolox equivalents/mg tissue.

#### 2.6.4. Reduced and Oxidised Glutathione Determination

Rat liver tissue lysates were used to measure total reduced glutathione (rGSH) and oxidised glutathione (GSSG) with a colorimetric assay kit (Sigma-Aldrich, St. Louis, MO, USA). GSH measurement relies on the glutathione recycling system, with GSH and DTNB fluorophore present. When DTNB undergoes reduction, it generates a stable fluorescent substance that can be detected spectrophotometrically. According to the manufacturer’s instructions, GSSG can be measured using a specific protocol that initially scavenges all existing GSH with 1-methyl-2-vinylpyridinium triflate as a scavenger reagent. Absorbance can be measured using an Infinite 200Pro plate reader (Tecan, Männedorf, Zurich, Switzerland). Final results were expressed as the ratio GSSG/total GSH (tGSH).

#### 2.6.5. Measurement of Superoxide Dismutase Activity (SOD; EC 1.15.1.1)

Total superoxide dismutase activity (SOD) in rat liver homogenates was measured by the SOD activity assay kit (Sigma-Aldrich, St. Louis, MO, USA). This method creates superoxide anions by the xanthine–xanthine oxidase system. Consequently, the superoxide anions reduce WST-1 to form WST-1 formazan, the absorbance of which was measured using an Infinite 200Pro plate reader (Tecan, Männedorf, Zurich, Switzerland). In the presence of SOD, O_2_^−^ underwent a dismutation process to produce O_2_ and H_2_O_2_, which resulted in a depletion of WST-1 formazan formation. The SOD activity (U/mL) was determined according to the manufacturer’s instructions using the inhibition curve.

#### 2.6.6. Measurement of Catalase Activity (CAT; EC 1.11.1.6)

Homogenised rat liver samples were used to determine catalase (CAT) activity according to Aebi [20]. Briefly, the reaction was performed in a total volume of 250 µL with 90 mM potassium phosphate buffer (pH 6.8), beginning with the addition of H_2_O_2_ (37.5 mM final concentration). Spectrophotometric measurement was then used to assess the disappearance of H_2_O_2_ at 240 nm. The results were reported as nmol/min.µg protein.

#### 2.6.7. Measurement of Glutathione Peroxidase Activity (GPx; EC 1.11.1.9)

Glutathione peroxidase (GPx) activity was assessed by determining the H_2_O_2_ scavenging capacity using the GPx assay kit (Sigma-Aldrich, St. Louis, MO, USA). GSSG was formed when H_2_O_2_ was reduced by GPx, and it was recycled in its reduced form (GSH) by both glutathione reductase (GR) and reduced nicotinamide adenine dinucleotide phosphate (NADPH). The decrease in NADPH absorbance was then measured using an Infinite 200Pro plate reader (Tecan, Männedorf, Zurich, Switzerland), with GPx activity expressed as U/L. µg protein.

### 2.7. Measurement of the Parameters Related to Inflammation, DNA Damage and Cell Death by Immunoblotting

NLR family pyrin domain containing 3 (NLRP3), Caspase-1, Interleukin 1 beta (IL-1β), Interleukin 6 (IL6), tumour necrosis factor alpha (TNF-α), phosphorylated-p38 MAPK, p38 mitogen-activated protein kinases (p38 MAPK), gamma-H2AX (γ-H2AX), H2A histone family member (H2AX), phosphorylated-ATM (Ser 1981), ATM serine/threonine kinase (ATM), Caspase-3 and Glyceraldehyde 3-phosphte dehydrogenase (GAPDH) proteins were measured.

Immunoblot analyses were carried out by loading 60 µg of total protein from liver extracts, which were denaturalised for 5 min at 95 °C in Laemmli Buffer [21] and separated through electrophoresis in either 4–15% (NLRP3, Caspase-1, IL-1β, IL6, TNF-α, p-p38 MAPK, p38 MAPK, γ-H2AX, H2AX, Caspase-3 and GAPDH) or 7.5% (p-ATM and ATM) SDS-polyacrylamide gels. The proteins were then transferred onto polyvinylidene difluoride (PVDF) membranes (Merck, Darmstadt, Germany) and blocked with 4% BSA for 1.5 h at room temperature. Next, they were blotted overnight at 4 °C with the appropriate antibodies: NLRP3 (1:1000; Abcam, Cambridge, UK), Caspase-1 (1:1000; Cell Signaling, Danvers, MA, USA), IL-1β (1:1000; Abcam, Cambridge, UK), IL6 (1:1000; Santa Cruz Biotech, Dallas, TX, USA), TNF-α (1:1000; Cell Signaling, Danvers, MA, USA), p-p38 MAPK (1:1000; Cell Signaling, Danvers, MA, USA), p38 MAPK (1:1000; Cell Signaling, Danvers, MA, USA), γ-H2AX (1:1000; Abcam, Cambridge, UK), H2AX (1:1000; Cell Signaling, Danvers, MA, USA), p-ATM (1:500; Novus Biologicals, Centennial, CO, USA), ATM (1:500; Abcam, Cambridge, UK), Caspase-3 (1:1000; Abcam, Cambridge, UK) and GAPDH (1:1000; Abcam, Cambridge, UK). Subsequently, membranes were incubated for 2 h at room temperature with polyclonal anti-mouse antibody (1:5000) (Santa Cruz Biotech, Dallas, TX, USA) for IL-6, γ-H2AX, p-ATM, Caspase-3 and GAPDH, and anti-rabbit antibody (1:5000) (Santa Cruz Biotech, Dallas, TX, USA) for NLRP3, Caspase-1, IL-1β, TNF-α, p-p38 MAPK, p38 MAPK, H2AX and ATM. The bound antibodies were visualised by an ECL system (Thermo Fisher Scientific Inc., Rockford, IL, USA) and quantified by a ChemiDoc MP Imaging System (Bio-Rad, Hercules, CA, USA). The measurements were normalised either by GAPDH or the phosphorylated isoforms.

### 2.8. Statistical Analysis

Results are presented as mean ± SEM. Statistical analysis was performed using SPSS 24.0 (SPSS, Chicago, IL, USA). The normal distribution of the data was confirmed by Shapiro–Wilks test. One-way ANOVA, followed by Newman–Keuls *post hoc* test, was used to compare the rats treated with extracts from each *Opuntia* species, the HFHF group and the control group. Significance was established at the *p* < 0.05 level.

## 3. Results

### 3.1. Body and Liver Weights, Hepatic Triglyceride Content, Serum Transaminase, Uric Acid and C-Reactive Protein Levels

At the end of the experimental period (8 weeks), the body weight, hepatic TG and serum parameters were determined in this in vivo model of non-alcoholic steatohepatitis. Compared to the control (C) group, rats in the high-fat high-fructose (HFHF) group presented a significantly higher body weight. Animals on the same steatotic diet and treated with either L-OD, H-OD, L-OFI or H-OFI did not show notable differences in this parameter (Table 3). High-fat high-fructose feeding led to an increase in energy intake that was not modified by *Opuntia* extracts. A similar trend was observed for liver weight, with all groups fed the high-fat high-fructose diet showing a significantly higher liver weight compared to the C group (Table 3).

With regard to hepatic TG, the HFHF group exhibited significantly higher levels compared to the C group, indicating the development of liver steatosis. The administration of L-OFI treatment partially reduced the liver TG levels (−12.5%) compared to the HFHF group (*p* < 0.05). For H-OFI, a tendency towards lower TG levels (−8.9%) in comparison to HFHF animals was found, whereas no differences in this parameter were observed with both OD extracts (Table 3). Concerning serum transaminases, the ALT and AST levels were significantly higher in the HFHF group compared to the C group. None of the treatments tested for ALT showed significant differences compared to animals in the HFHF group. Nonetheless, a significant decrease in serum AST levels was observed with the administration of H-OFI compared to the HFHF group (*p* < 0.05), albeit the decrease in this group did not reach the levels seen in the C group (Table 3).

Moreover, although the steatotic diet did not significantly boost the serum uric acid levels, a notable reduction in this parameter was observed following H-OD supplementation compared to the HFHF group (*p* < 0.05) (Table 3). The serum CRP levels, an inflammatory biomarker, showed a substantial rise in the HFHF group (+142.98) compared to the C group. All treatments significantly prevented this diet-induced elevation, resulting in even lower levels than those observed in animals on the standard diet (*p* < 0.05). Notably, animals supplemented with H-OD and H-OFI exhibited significantly lower levels of this parameter compared to the C group (*p* < 0.05), with reductions of −47.8% and −84.7%, respectively (Table 3).

### 3.2. Parameters Related to Oxidative Stress in Liver

**Figure 1:**
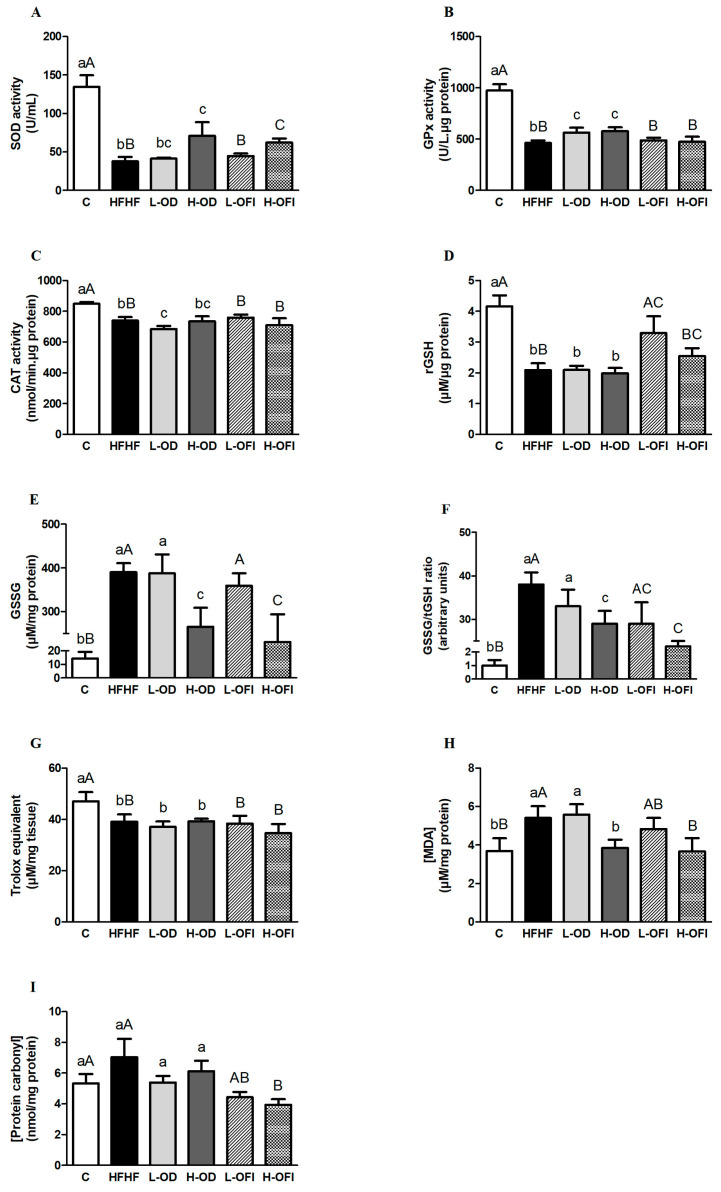
Activity of antioxidant enzymes SOD (**A**), GPx (**B**) and CAT (**C**), and content of rGSH (**D**), GSSG (**E**), GSSG/tGSH ratio (**F**), antioxidant capacity (ORAC assay, (**G**)), and levels of MDA (**H**) and protein carbonyl content (**I**) in liver samples from rats fed a control diet (**C**), a high-fat high-fructose diet (HFHF) or a high-fat high-fructose diet supplemented with low or high doses of *Opuntia stricta* var. *dillenii* peel extract (L-OD and H-OD, respectively) or the same diet supplemented with low or high doses of *Opuntia ficus-indica* var. *colorada* pulp extract (L-OFI and H-OFI, respectively). Values are represented as mean ± SEM. Differences among groups were established using one-way ANOVA, followed by the Newman–Keuls *post hoc* test. Bars not sharing common letters are significantly different (*p* < 0.05). Lowercase letters represent differences among C, HFHF, L-OD and H-OD groups, and uppercase letters show differences among C, HFHF, L-OFI and H-OFI groups. CAT: catalase; GPx: glutathione peroxidase; GSSG: oxidised glutathione; MDA: malondialdehyde; SOD: superoxide dismutase; rGSH: reduced glutathione; tGSH: total glutathione.

In order to assess the antioxidant effects of the treatments on the hepatic oxidative stress induced by a high-fat high-fructose diet, the activity of key enzymes involved in maintaining redox homeostasis, levels of nonenzymatic antioxidants and protein and lipid peroxidation products were measured. The antioxidant enzyme activity analysis showed that animals in the HFHF group had significantly lower SOD activity (*p* < 0.001), in addition to a significantly decreased GPx activity when compared to the C group (*p* < 0.001), as shown in Figure 1A,B. Moreover, CAT activity was also suppressed in the HFHF group (*p* < 0.001) (Figure 1C). Regarding the parameters of the non-enzymatic antioxidant system, the GSSG levels were substantially boosted in the HFHF group compared to controls (*p* < 0.001), while the steatotic diet caused a significant decrease in the rGSH content (*p* < 0.001). These results, consequently, led to an increase in the ratio GSSG/tGSH in the HFHF group compared to the control group (*p* < 0.001), as shown in Figure 1D–F. As for the total antioxidant capacity (ORAC), a reduction was noted when comparing to the C group (*p* < 0.05; Figure 1G). Concerning the lipid peroxidation product, a significant increase in the hepatic MDA content was observed in the HFHF group compared to the C group (*p* < 0.05; Figure 1H). However, no significant differences were noticed in the protein carbonyl content between the HFHF and C groups (*p* = 0.14; Figure 1I).

Regarding the effects of the *Opuntia* extracts on these parameters, supplementation with H-OD and H-OFI significantly enhanced the SOD activity (86.6%, *p* < 0.05 and 63.9%, *p* < 0.01, respectively) compared to the HFHF group, while rats in the L-OD and H-OFI groups did not show an increase. Concerning the GPx activity, the L-OD and H-OD groups avoided the decrease caused by the steatotic diet (*p* < 0.05), whereas neither L-OFI nor H-OFI treatments modified this parameter compared to the HFHF group. Lastly, none of the tested treatments augmented the CAT activity reduced by the HFHF diet. As for the non-enzymatic antioxidants, the H-OD and H-OFI groups significantly avoided the increase in GSSG content observed in the HFHF group (*p* < 0.01 and *p* < 0.05, respectively); by contrast, no changes were noted with both L-OD and L-OFI groups. For the rGSH levels, animals in the L-OFI group showed a significant increase in this parameter compared to those in the HFHF group (*p* < 0.05), while the H-OFI supplementation group exhibited an upward trend (*p* = 0.1). The other treated groups, L-OD and H-OD, did not show any differences compared to the HFHF group regarding this parameter. Overall, the treatments that significantly improved the elevated ratio of GSSG/tGSH caused by the steatotic diet were H-OD and H-OFI (*p* < 0.05 and *p* < 0.01, respectively). Following supplementation with L-OFI, a downward trend in the ratio was observed when compared to the HFHF group (*p* = 0.08), and no differences were found in the L-OD group. In addition, none of the tested doses or extracts prevented the decrease in the total antioxidant capacity observed in the HFHF group.

Concerning lipid peroxidation, treatment with both H-OD and H-OFI prevented the boost in MDA content caused by HFHF feeding by −28.7% and −32.1%, respectively (*p* < 0.05). The administration of low doses of both extracts to the L-OD and L-OFI groups did not show differences compared to either HFHF or C groups for this parameter. Although the protein carbonyl content was not affected by the steatotic diet, rats in the L-OFI and H-OFI groups exhibited a significant reduction in this parameter compared to the HFHF group (*p* < 0.05). Indeed, lower values of protein carbonyl content were observed in animals treated with H-OFI compared to the C group (*p* = 0.05 vs. C).

### 3.3. Parameters Related to Inflammation in Liver

**Figure 2:**
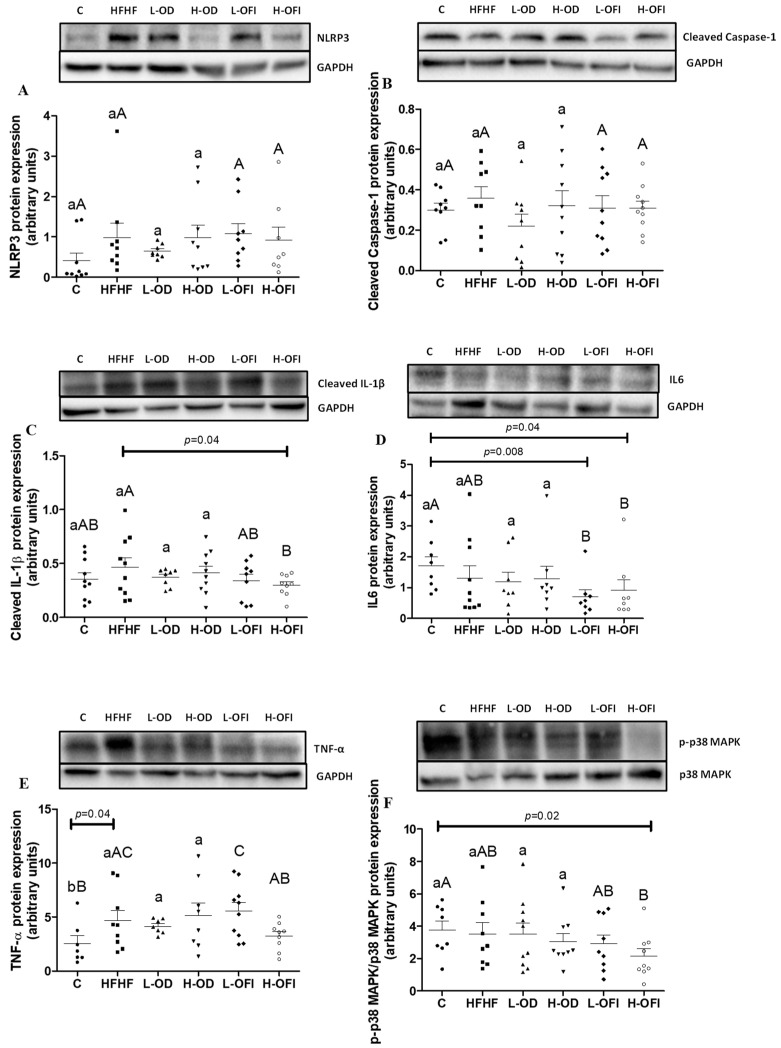
Hepatic protein expression levels of NLRP3 (**A**), cleaved Caspase-1 (**B**), mature IL-1β (**C**), IL6 (**D**), TNF-α (**E**) and activation rate (phosphorylation) of p38 MAPK (**F**) in liver samples from rats fed a control diet (**C**), a high-fat high-fructose diet (HFHF) or a high-fat high-fructose diet supplemented with low or high doses of *Opuntia stricta* var. *dillenii* peel extract (L-OD and H-OD, respectively) or the same diet supplemented with low or high doses of *Opuntia ficus-indica* var. *colorada* pulp extract (L-OFI and H-OFI, respectively). The western blot bands shown are representative of 10 samples/groups. Values are represented as mean + SEM. Differences among groups were established using one-way ANOVA, followed by the Newman–Keuls *post hoc* test. Groups not sharing common letters are significantly different (*p* < 0.05). Lowercase letters represent differences among C, HFHF, L-OD and H-OD groups, and uppercase letters show differences among C, HFHF, L-OFI and H-OFI groups. IL-1β: interleukin 1 beta; IL6: interleukin 6; NLRP3: NLR family pyrin domain containing 3; p38 MAPK: p38 mitogen-activated protein kinases; TNF-α: tumour necrosis factor alpha.

Inflammation plays a key role in the development of MAFLD, and in the past decade, several reports have demonstrated an association between the NLRP3 inflammasome activation and MAFLD pathogenesis [22]. Taking this into account, the protein expression of NLRP3, Caspase-1, IL-1β, IL6, TNF-α and p38 MAPK in the liver was measured. Regarding the NLRP3 and Caspase-1 levels, no statistically significant differences were observed in the HFHF group compared to the C group (Figure 2A,B). Concerning IL-1β, IL6 and p38 MAPK phosphorylation, no differences were observed between the HFHF and C groups (Figure 2C,D,F). Nevertheless, a sharp increase was noted in TNF-α protein expression in the HFHF group (*p* < 0.05; Figure 2E).

Regarding the inflammasome-related proteins, none of the treatments showed to be significantly different compared to the HFHF group or to the controls. Concerning IL-1β, animals in the H-OFI group showed significantly reduced values compared to the HFHF group (*p* < 0.05). Regarding IL6, while no significant difference was observed between the HFHF and C groups, both L-OFI and H-OFI groups displayed a notable reduction compared to the C group (*p* < 0.01 and *p* < 0.05, respectively), albeit OD extract did not improve this parameter. For TNF-α levels, this parameter remained unchanged in all the treated animals compared to the HFHF group. In the case of p-p38 MAPK protein levels, H-OFI supplementation was shown to significantly decrease this parameter compared to the C group (*p* < 0.05). In this instance, the other treatments did not reduce the p-p38 MAPK levels.

### 3.4. Parameters Related to DNA Damage and Cell Death in Liver

**Figure 3:**
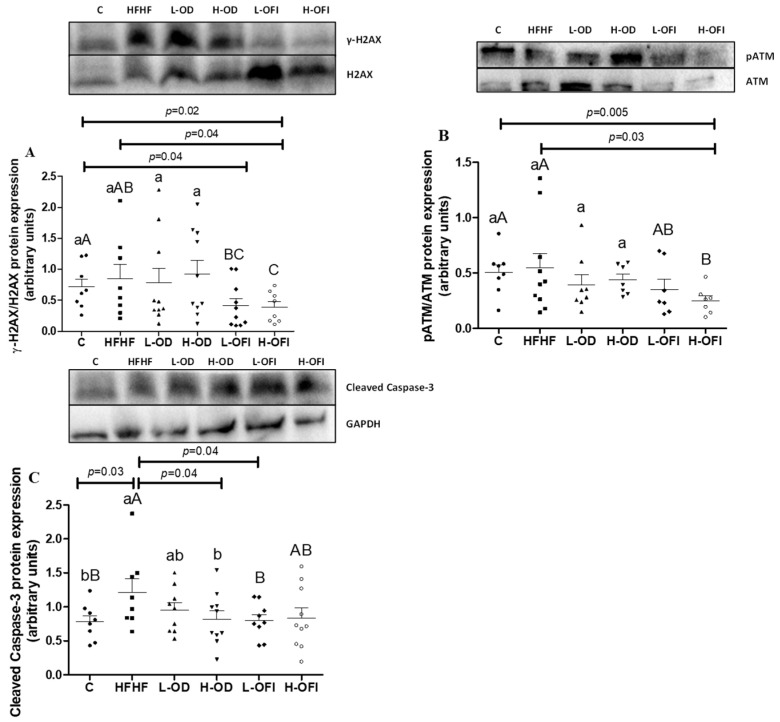
Activation rate of H2AX (**A**), ATM (**B**) and protein expression levels of Caspase-3 (**C**) in liver samples from rats fed a control diet (**C**), a high-fat high-fructose diet (HFHF) or a high-fat high-fructose diet supplemented with low or high doses of *Opuntia stricta* var. *dillenii* peel extract (L-OD and H-OD, respectively) or the same diet supplemented with low or high doses of *Opuntia ficus-indica* var. *colorada* pulp extract (L-OFI and H-OFI, respectively). The western blot bands shown are representative of 10 samples/groups. Values are represented as mean + SEM. Differences among groups were established by using one-way ANOVA, followed by the Newman–Keuls *post hoc* test. Groups not sharing common letters are significantly different (*p* < 0.05). Lowercase letters represent differences among C, HFHF, L-OD and H-OD groups, and uppercase letters show differences among C, HFHF, L-OFI and H-OFI groups. ATM: ATM serine/threonine kinase; H2AX: H2A histone family member.

Given the well-known damage to DNA and cell viability caused by oxidative stress and inflammatory conditions, some related markers were analysed. For the phosphorylated forms of H2AX and ATM proteins, no significant difference was observed between the HFHF and the C groups (Figure 3A,B). In contrast, a significant increase of 56.1% in the Caspase-3 levels was noted in the HFHF group compared to the controls (*p* < 0.05; Figure 3C).

Analysis of the different treatments revealed a significant decrease in the γ-H2AX levels in the H-OFI group compared to both the HFHF and the C groups (*p* < 0.05). Moreover, a significant reduction was noted with the L-OFI treatment compared to the C group (*p* < 0.05) (Figure 3A). Rats in the L-OD and H-OD groups did not differ from those in the HFHF group with respect to this parameter. A similar pattern was observed for the p-ATM protein expression, with the H-OFI group showing significantly reduced levels compared to both the HFHF (*p* < 0.05) and the C groups (*p* < 0.01; Figure 3B), while the other treatments did not alter the p-ATM values. Lastly, Caspase-3 levels were significantly decreased in animals from both the H-OD and L-OFI groups (*p* < 0.05). The L-OD and H-OFI groups did not show a significant difference from the HFHF group for this parameter.

## 4. Discussion

**Figure 4:**
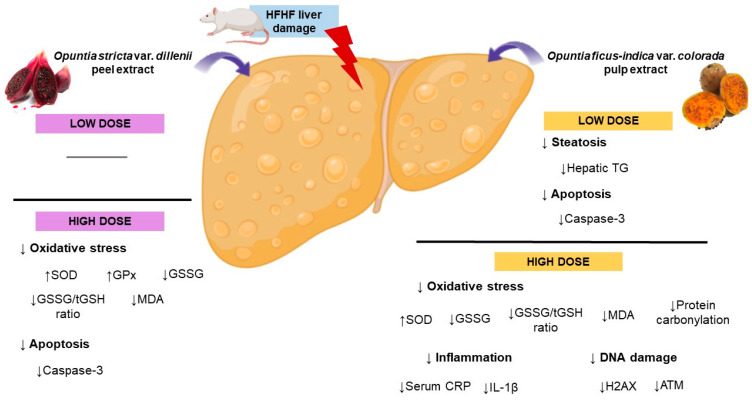
Summary of the beneficial effects of each treatment on the prevention of diet-induced MAFLD. ATM: ATM serine/threonine kinase; CRP: C-reactive protein; GPx: glutathione peroxidase; GSSG: oxidised glutathione; H2AX: H2A histone family member; HFHF: high-fat high-fructose; IL-1β: interleukin 1 beta; MDA: malondialdehyde; SOD: superoxide dismutase; TG: triglyceride; tGSH: total glutathione.

High fructose intake is a well-known risk factor for the development of MAFLD. The negative impact of dietary fructose on health is most pronounced when consumed alongside a high-fat diet [4]. Indeed, excessive intake of fructose leads to hepatic TG accumulation, oxidative stress and inflammation, among other effects [23]. Significant attention has been directed toward plant-derived compounds to discover new tools for preventing or managing metabolic alterations. Since bioactive compounds present in *Opuntia* fruits, such as betalains and phenolic compounds, exhibit radical scavenging properties [24,25], the aim of our study was to analyse the effects of two different *Opuntia* varieties on oxidative stress and inflammation, both of which are associated with steatosis and its progression to steatohepatitis. Each administered treatment has demonstrated benefits for certain complications related to MAFLD. The deviations in the observed effects were likely due to variations in the content of bioactive compounds in each extract and the different doses used (Figure 4).

Based on the results described in the present study, the supplementation with *Opuntia stricta* var. *dillenii* peel extract showed no changes in hepatic TG content. These findings cannot be compared to the existing literature, as no data have been reported thus far. Considering that *Opuntia stricta* var. *dillenii* is characterised by a high betanin content, the most abundant betacyanin, it would be valuable to analyse the effects observed in studies involving these compounds. In this regard, Song et al. [26] reported that a high-fat diet supplemented with a purified pitaya peel betacyanin extract was able to significantly reduce the hepatic TG levels in a dose-dependent manner. The discordance with our findings may be due to the fact that the extract used by Song et al. had a higher betacyanin content, resulting in the rats receiving a higher amount of these compounds with this treatment.

Serum transaminase levels are commonly used as markers of liver damage. In this regard, the HFHF diet led to a significant rise in both the serum ALT and AST levels, consistent with most studies using similar experimental models [27,28,29]. None of the doses of *Opuntia stricta* var. *dillenii* peel extract were able to significantly reduce the serum ALT or AST levels. This fact is probably related to the lack of improvement in steatosis induced by *Opuntia stricta* var. *dillenii.*

With respect to hepatic TG content, the low dose of *Opuntia ficus-indica* var. *colorada* pulp extract significantly reduced this parameter, while a tendency toward lower values was observed with a high dose of the extract. This hepatic lipid-lowering effect has also been observed in other studies involving extracts of this *Opuntia* species obtained from other parts of the plant, such as the study reported by Morán-Ramos et al. [30] investigating Zucker rats fed a standard diet and treated with an *Opuntia ficus-indica* cladode extract, or the study by Kang et al. [31] in C57BL7/6 mice fed a high-fat diet and supplemented with an *Opuntia ficus-indica* DWJ504 seed extract. The fact that higher doses of *Opuntia* extracts exhibit lesser effects than lower doses has also been observed by other authors. For instance, Kang et al. tested different doses of the DWJ504 seed extract, finding that the highest dose was not the most effective in reducing the hepatic TG content.

Concerning the serum transaminases levels, a significant reduction in AST was observed with the high-dose supplementation of *Opuntia ficus-indica* var. *colorada* pulp extract. The results of the present research align with those reported by Bouazza A et al. [32], who noted a significant reduction in the serum AST levels but not in serum ALT levels following *Opuntia ficus-indica* vinegar supplementation in Wistar rats. Other studies have reported a significant decrease in the serum ALT but not in AST levels in mice fed a high-fat diet and treated with 250, 500 or 1000 mg of *Opuntia ficus-indica* DWJ504 seed extract/kg body weight/day for four weeks [31]. This difference may be due to the animal model used or the cactus part. In summary, our study indicates that *Opuntia ficus-indica* var. *colorada* pulp extract appears to be the most effective at preventing hepatic steatosis, with the lower dose showing a higher anti-steatotic effect. The different effectiveness of both extracts may be related to their distinct bioactive compound compositions, as described in the Materials and Methods section.

In the presence of excessive hepatic lipid accumulation, mitochondrial dysfunction and impaired electron transport chain function cause RONS production [9]. As widely recognised, oxidative stress refers to an imbalance between the generation of RONS and the scavenging capacity of the host in favour of the former [5]. This oxidative stress has been considered a key factor in liver injury and disease progression. Therefore, the main parameters related to oxidative stress and the end products of lipid and protein oxidation were measured to assess the antioxidant capacity of *Opuntia* extracts. In our experiment, the RONS scavenging capacity of rats fed the HFHF diet was reduced, as the enzymatic activities of SOD, CAT and GPx were significantly lower compared to the control group. Moreover, the hepatic non-enzymatic antioxidant rGSH content was significantly decreased, while the GSSG levels were boosted in rats fed the steatotic diet, resulting in a significant enhancement of the GSSG/tGSH ratio in this group. These data are in good accordance with the observed reduction in total antioxidant capacity assessed by ORAC. In addition, the obtained results align with previously described effects of high fructose consumption on liver oxidative stress [23,33]. The imbalance between the increased RONS and decreased antioxidants results in lipid peroxidation of polyunsaturated fatty acids, generating cytotoxic aldehydes, such as MDA [34], as observed in the present study. Under prolonged or elevated oxidative stress, the reactive lipid aldehydes induce the posttranslational modification of cysteine, histidine and lysine residues of proteins through a process known as protein carbonylation [35]. In this context, an increase in protein carbonyl levels was also observed in the group of rats fed a steatotic diet, although this rise did not reach statistical significance. This may be due to the fact that although the animals are experiencing oxidative stress, a longer experimental period may be required to significantly increase this parameter.

Antioxidants have been commonly recognised for their ability to act as scavengers of free radicals, effectively diminishing the damage caused by oxidation in the liver [36,37]. Although *Opuntia* species are rich in bioactive compounds exhibiting antioxidant properties, such as flavonoids, phenolic compounds and betalains, the literature analysing this effect is scarce, with *Opuntia ficus-indica* being the most studied species [12]. In our experiment, the high dose of *Opuntia stricta* var. *dillenii* peel extract increased the SOD activity and prevented the decrease in the GPx activity induced by the HFHF diet. We also observed a significant reduction in GSSG levels with this treatment, resulting in a lowered GSSG/tGSH ratio. Consequently, the high dose of *Opuntia stricta* var. *dillenii* peel extract led to a significant decrease in lipid peroxidation, as determined by the MDA content. Zhu X et al. [38] also reported a boost in the SOD and GPx activities, consistent with our results. Furthermore, other studies involving *Opuntia stricta* var. *dillenii* have demonstrated a decrease in the hepatic MDA content [38,39,40,41]. Uric acid, a by-product of fructose metabolism, is considered a mediator of the hepatotoxic effects of this sugar and is involved in oxidative stress [42]. In the present study, although the administration of a high dose of *Opuntia stricta* var. *dillenii* peel extract reduced serum uric acid levels (−12%) compared to the HFHF group, this difference did not reach statistical significance, suggesting that under our experimental conditions, uric acid does not appear to be involved in the oxidative stress induced by the HFHF diet.

Concerning *Opuntia ficus-indica* var. *colorada* pulp extract, the high dose increased the SOD activity and decreased the GSSG levels, leading to a significant reduction in the GSSG/tGSH ratio compared to the HFHF group. The low dose of this extract significantly augmented rGSH levels. These results are in accordance with those reported by Bouazza A et al. [32], who observed an increase in SOD and GPx activities in rats fed a high-fat diet supplemented with the cactus extract. Kang J et al. [31] also observed an increase in the hepatic rGSH content with *Opuntia ficus-indica* treatment in rats fed a high-fat diet. Other studies analysing liver damage induced by chemicals and supplementation with *Opuntia ficus-indica* juice or cladode extract have also reported an increase in SOD, CAT and GPx activities [43,44]. In our study, a reduction in the hepatic MDA levels was also observed in animals treated with a high dose of *Opuntia ficus-indica* var. *colorada* pulp extract, resulting from an improvement in the oxidative stress-related parameters. This result is in line with other studies using *Opuntia ficus-indica* cladode juice [45], fruit juice [43] or cladode extract [46,47], all of which reported a decrease in hepatic MDA content.

Overall, high doses of *Opuntia stricta* var. *dillenii* peel extract and *Opuntia ficus-indica* pulp extract appear to be the most effective treatments for preventing oxidative stress induced by a steatotic diet, with *Opuntia stricta* var. *dillenii* showing slightly better results. This is attributed to the H_2_O_2_ content generated after SOD activity being converted to water due to the upregulation of GPx.

Systemic inflammation plays a key role in the progression of simple liver steatosis to steatohepatitis and fibrosis [48]. In this context, the HFHF diet in our study led to higher levels of serum CRP, which is an inflammatory mediator. These results are in line with those reported in other studies using similar experimental models [49]. There is increasing evidence that the NLRP3 inflammasome plays an important role in both inflammation and fibrosis in metabolic syndrome [50]. NLRP3 inflammasomes are cytosolic supramolecular complexes that activate Caspase-1, which processes the IL-1β and interleukin-18 (IL-18) to their mature forms [51]. Furthermore, lipotoxicity induced by excessive fat accumulation in hepatocytes leads to the activation of Kupffer cells, promoting the production of proinflammatory cytokines, such as IL-6, TNF-α and IL-1β [50]. Mitogen-activated protein (MAP) kinases play key roles in responding to different external signals, with the activation of the p38 pathway being crucial for the synthesis of the aforementioned proinflammatory cytokines [52]. In the present study, the measurement of key inflammasome proteins in the liver showed that the HFHF diet did not activate the NLRP3/Caspase-1/IL-1 β pathway. Regarding hepatic IL-6 protein expression, no significant difference was observed between the HFHF and control groups. This may be explained by the lack of p38 MAPK activation in these rats compared to the control group. The effectiveness of the diet in generating inflammation could also be determined by analysing TNFα, since this marker is a key trigger of inflammation in the development of metabolic steatohepatitis [53]. In the present study, hepatic TNF-α levels were significantly higher in rats fed the steatotic diet. Overall, these results indicate that the HFHF diet administered was effective in inducing inflammation by up-regulating the TNF-α pro-inflammatory cytokine in these animals.

In regard to treatment with *Opuntia stricta* var. *dillenii* peel extract, both doses significantly decreased the serum CRP levels. Taking into account that CRP levels increase when there is systemic inflammation and considering that the markers related to inflammation in the liver have not been modified by this *Opuntia* extract, we can suggest that the CRP decrease in these animals may be due to the inflammation prevention in other organs that have not been analysed in the present study.

*Opuntia ficus-indica* var. *colorada* pulp extract, at both doses, decreased CRP serum levels, with the high dose demonstrating the most significant anti-inflammatory activity, as this parameter was even lower in this group than in the control group. In contrast, the only study reported in the literature with *Opuntia* species that measured the serum CRP levels found no significant difference between rats fed a high-fat diet supplemented with *Opuntia ficus-indica* cladode extract and those fed the same diet without treatment [54]. This discrepancy may be attributed to differences in the composition of the extract administered or the animal model used. In the present study, the high dose of *Opuntia ficus-indica* var. *colorada* pulp extract also significantly decreased the cleaved IL-1β and IL-6 levels compared to rats fed a steatotic diet or the control group, respectively. In line with these results, rats fed with the high dose of *Opuntia ficus-indica* var. *colorada* pulp extract showed reduced p38 MAPK activation compared to the control group. Summarising, TNF-α, which is the only inflammatory parameter modified by HFHF feeding, was not affected by *Opuntia ficus-indica* var. *colorada* extracts. Moreover, although the high dose of this *Opuntia* species did reduce IL-1β, IL-6 and p38 MAPK activation, in fact the values of these parameters found in all groups fed the HFHF diet were physiological because no significant differences were found with the control group. Consequently, a clear anti-inflammatory effect of the *Opuntia ficus-indica* var. *colorada* extracts at the hepatic level cannot be proposed in the frame of this experimental design.

Hepatic inflammation is established as a major risk factor in the development of HCC due to its implication in oxidative DNA damage [55,56]. Consequently, the state of inflammation, DNA damage or excessive RONS production can induce apoptosis, which appears to be the primary mechanism of cell death in metabolic syndrome [57]. Apoptosis can elicit the activation of fibrogenesis, and it is considered a crucial event in the progression of MAFLD to more detrimental stages of liver disease, such as HCC [57,58]. In the present study, the protein expression of phosphorylated ATM and H2AX was measured as markers of liver DNA damage. In this process, double-stranded breaks (DSB), which are deleterious forms of DNA damage, are generated. Therefore, the activation of the DSB repair pathway occurs, which is partially regulated by ATM, leading to the activation of H2AX by phosphorylation (γ-H2AX) to facilitate the repair [55]. Activated Caspase-3 is located at the final stage of the Caspase cascades, triggered by both endogenous and exogenous apoptotic pathways, and is considered a crucial protein for apoptosis and pyroptosis, a newly identified inflammatory form of cell death [59]. In the present experiment, the HFHF diet did not induce a significant increase in p-ATM and γ-H2AX, indicating that nucleic acid damage was not induced by the steatotic diet over the experimental period of 8 weeks. Similar results have been previously reported in rodent studies over a period of six to eight weeks [29,60]. Regarding Caspase-3, rats fed the steatotic diet showed higher levels of this protein expression compared to the control group, indicating the activation of the apoptotic pathway.

In the case of supplementation with a high dose of *Opuntia stricta* var. *dillenii* peel extract, the treatment completely prevented the increase in Caspase-3 levels, which returned to control values, probably due to the reduction of oxidative stress in this group, as mentioned above.

With regard to the *Opuntia ficus-indica* var. *colorada* pulp extract, a significant decrease in the p-ATM and γ-H2AX parameters was observed with the high dose administration, compared to both the HFHF and the control groups. Furthermore, the treatment with the low dose of *Opuntia ficus-indica* var. *colorada* pulp extract also significantly reduced Caspase-3 levels, similar to the prevention of TG accumulation associated with this extract. In this context, lipotoxicity has been identified as a key trigger of apoptosis [61]. Specifically, it has been demonstrated that certain cytokines, such as interleukin 11, are secreted by lipotoxic hepatocytes to induce Caspase-3 activation [62]. Lastly, a high dose of the same extract resulted in a decrease in the expression of this protein compared to the HFHF group, likely due to the efficacy of this treatment in improving inflammatory and DNA damage markers.

## 5. Conclusions

In conclusion, although only the low dose of *Opuntia ficus-indica* var. *colorada* pulp extract was able to reduce hepatic triglyceride accumulation, both *Opuntia* species extracts are effective in regulating the progression to metabolic steatohepatitis through different mechanisms of action. Specifically, the *Opuntia ficus-indica* var. *colorada* pulp extract at the high dose decreases the non-enzymatic antioxidant system (GSSG/tGSH ratio), thereby preventing lipid peroxidation, and reduces the expression of DNA damage markers. Moreover, at low dose, this extract is able to prevent the cell death induced by HFHF feeding. Regarding *Opuntia stricta* var. *dillenii* peel extract, it prevents oxidative stress and apoptosis only at the high dose.

## Figures and Tables

**Table 1:** Quantification of the main phenolic compounds and betalains present in *Opuntia ficus-indica* var. *colorada* pulp extract and *Opuntia stricta* var. *dillenii* peel extract by HPLC–DAD–MS.

Compound	Content (µg of Compound/g Dry Weight)	
	*Opuntia stricta* var. *dillenii* Peel Extract	*Opuntia ficus-indica* var. *colorada* Pulp Extract
Portulacaxanthin III (Bx-glycine)	n.d.	30 ± 1.6
Vulgaxanthin III (Bx-asparagine)	n.d.	14.6 ± 0.9
Vulgaxanthin I (Bx-glutamine)	n.d.	12.4 ± 0.4
Vulgaxanthin II (Bx-glutamic acid)	n.d.	18 ± 2.9
Indicaxanthin (Bx-proline)	n.d.	510 ± 14
Betanin	5160 ± 88	tr.
Isobetanin	3038 ± 95	n.d.
2′-O-apiosyl-4-O-phyllocactin	1372 ± 102	n.d.
**5** **″** **-O-E-sinapoyl-2-apiosyl-phyllocactin**	183 ± 37	n.d.
Neobetanin	429 ± 11	n.d.
Piscidic acid	19,269 ± 382	2564 ± 108
**Quercetin-3-O-rhamnosyl-rutinoside (QG3)**	202 ± 6	n.d.
Quercetin glycoside 2 (QG2)	245 ± 9	14.4 ± 3.3
Isorhamnetin glucoxyl-rhamnosyl-pentoside (IG2)	989 ± 21	30.4 ± 3.9

n.d. not detected; tr.: traces.

**Table 2:** Nutritional composition of experimental diets.

	STD	HFHF
Total energy (kcal/g)	3.9	4.5
Carbohydrates (energy %)	63.9	40
Fructose (energy %)	-	10
Proteins (energy %)	20.3	20
Lipids (energy %)	15.8	40

g: grams; HFHF: high-fat high-fructose; kcal: kilocalories; STD: Standard diet.

**Table 3:** Final body weight, daily energy intake, liver weight, hepatic triacylglycerol (TG) content, serum alanine aminotransferase (ALT), serum aspartate aminotransferase (AST) levels, serum uric acid and serum C-reactive protein (CRP) levels of rats fed a standard diet (C) and a high-fat high-fructose diet alone (HFHF) or supplemented with low and high doses of *Opuntia stricta* var. *dillenii* peel extract (L-OD and H-OD, respectively) or with low and high doses of *Opuntia ficus-indica* var. *colorada* pulp extract (L-OFI and H-OFI, respectively) for 8 weeks.

	C	HFHF	L-OD	H-OD	L-OFI	H-OFI	ANOVA
Final body weight (g)	232.0 ± 10 ^bB^	281.0 ± 12 ^aA^	267.0 ± 14 ^a^	261.0 ± 13 ^a^	274.0 ± 11 ^A^	277.0 ± 14 ^A^	*p* < 0.05
Food intake (g/day)	19.3 ± 0.3 ^aA^	20.4 ± 0.4 ^aA^	20.1 ± 0.6 ^a^	19.5 ± 0.2 ^a^	19.6 ± 0.3 ^A^	20.1 ± 0.4 ^A^	NS
Energy intake (kcal)	76.6 ± 1.1 ^bB^	91.7 ± 1.8 ^aA^	90.0 ± 3.0 ^a^	88.0 ± 1.0 ^a^	88.0 ± 1.0 ^A^	90.0 ± 2.0 ^A^	*p* < 0.05
Liver weight (g)	10.7 ± 0.3 ^bB^	22.0 ± 0.9 ^aA^	20.7 ± 0.9 ^a^	20.8 ± 0.6 ^a^	20.6 ± 1.0 ^A^	21.0 ± 1.1 ^A^	*p* < 0.05
Liver TG (mg/g tissue)	20.0 ± 1.7 ^bB^	50.4 ± 2.5 ^aA^	46.8 ± 1.5 ^a^	47.0 ± 1.9 ^a^	44.1 ± 1.8 ^C^	45.9 ± 2.5 ^AC^	*p* < 0.05
ALT (U/L)	14.1 ± 0.7 ^bB^	100.1 ± 14.2 ^aA^	110.0 ± 12.6 ^a^	86.7 ± 11.0 ^a^	103.4 ± 13.5 ^A^	97.0 ± 15.1 ^A^	*p* < 0.05
AST (U/L)	75.8 ± 3.7 ^bB^	118.2 ± 10.8 ^aA^	129.0 ± 7.6 ^a^	112.4 ± 4.9 ^a^	112.5 ± 3.7 ^A^	93.4 ± 7.0 ^C^	*p* < 0.05
Uric acid (mg/dL)	3.2 ± 0.2 ^abA^	3.4 ± 0.1 ^aA^	3.6 ± 0.2 ^a^	3.0 ± 0.1 ^b^	3.3 ± 0.2 ^A^	3.3 ± 0.1 ^A^	*p* < 0.05
CRP (µg/mL)	271.4 ± 51.6 ^bB^	659.4 ± 270.2 ^aA^	162.3 ± 38.3 ^bc^	141.8 ± 37.1 ^c^	171.5 ± 47.4 ^B^	41.6 ± 6.4 ^C^	*p* < 0.05

Values are presented as mean ± SEM. Differences among groups were determined using a one-way ANOVA followed by the Newman–Keuls *post hoc* test. Values not sharing a common letter are significantly different (*p* < 0.05). Lowercase letters represent differences among C, HFHF, L-OD and H-OD groups, and uppercase letters show differences among C, HFHF, L-OFI and H-OFI groups.

## Data Availability

The raw data supporting the conclusions of this article will be made available by the authors on request.
